# The gut microbiome in bullous pemphigoid: implications of the gut-skin axis for disease susceptibility

**DOI:** 10.3389/fimmu.2023.1212551

**Published:** 2023-11-10

**Authors:** Xiaolin Liu, Nina van Beek, Aleksa Cepic, Nadia A. Andreani, Cecilia J. Chung, Britt M. Hermes, Kaan Yilmaz, Sandrine Benoit, Kossara Drenovska, Sascha Gerdes, Regine Gläser, Matthias Goebeler, Claudia Günther, Anabelle von Georg, Christoph M. Hammers, Maike M. Holtsche, Franziska Hübner, Dimitra Kiritsi, Franziska Schauer, Beke Linnenmann, Laura Huilaja, Kaisa Tasanen-Määttä, Snejina Vassileva, Detlef Zillikens, Christian D. Sadik, Enno Schmidt, Saleh Ibrahim, John F. Baines

**Affiliations:** ^1^ Department of Evolutionary Genetics, Max Planck Institute for Evolutionary Biology, Plön, Germany; ^2^ Section of Evolutionary Medicine, Institute for Experimental Medicine, Kiel University, Kiel, Germany; ^3^ Department of Dermatology, Allergy, and Venereology, University of Lübeck, Lübeck, Germany; ^4^ Department of Dermatology, Venereology and Allergology, University Hospital Würzburg, Würzburg, Germany; ^5^ Department of Dermatology and Venereology, Medical University-Sofia, Sofia, Bulgaria; ^6^ Department of Dermatology, Venereology and Allergology, University of Kiel, Kiel, Germany; ^7^ Department of Dermatology, University Hospital, Technische Universität (TU) Dresden, Dresden, Germany; ^8^ Department of Dermatology, Faculty of Medicine, Medical Center-University of Freiburg, Freiburg, Germany; ^9^ Research Unit of Clinical Medicine, University of Oulu, Oulu, Finland; ^10^ Department of Dermatology and Medical Research Center Oulu, Oulu University Hospital, Oulu, Finland; ^11^ Center for Research on Inflammation of the Skin (CRIS), University of Lübeck, Lübeck, Germany; ^12^ Lübeck Institute of Experimental Dermatology (LIED), University of Lübeck, Lübeck, Germany; ^13^ College of Medicine and Health Sciences, Khalifa University, Abu Dhabi, United Arab Emirates

**Keywords:** bullous pemphigoid, skin, inflammation, gut microbiome, 16S rRNA gene, metagenome

## Abstract

Bullous pemphigoid (BP) is an autoimmune blistering disease that primarily affects the elderly. An altered skin microbiota in BP was recently revealed. Accumulating evidence points toward a link between the gut microbiota and skin diseases; however, the gut microbiota composition of BP patients remains largely underexplored, with only one pilot study to date, with a very limited sample size and no functional profiling of gut microbiota. To thoroughly investigate the composition and function of the gut microbiota in BP patients, and explore possible links between skin conditions and gut microbiota, we here investigated the gut microbiota of 66 patients (81.8% firstly diagnosed) suffering from BP and 66 age-, sex-, and study center-matched controls (CL) with non-inflammatory skin diseases (132 total participants), using 16S rRNA gene and shotgun sequencing data. Decreased alpha-diversity and an overall altered gut microbial community is observed in BP patients. Similar trends are observed in subclassifications of BP patients, including first diagnoses and relapsed cases. Furthermore, we observe a set of BP disease-associated gut microbial features, including reduced *Faecalibacterium prausnitzii* and greater abundance of pathways related to gamma-aminobutyric acid (GABA) metabolism in BP patients. Interestingly, *F. prausnitzii* is a well-known microbiomarker of inflammatory diseases, which has been reported to be reduced in the gut microbiome of atopic dermatitis and psoriasis patients. Moreover, GABA plays multiple roles in maintaining skin health, including the inhibition of itching by acting as a neurotransmitter, attenuating skin lesions by balancing Th1 and Th2 levels, and maintaining skin elasticity by increasing the expression of type I collagen. These findings thus suggest that gut microbiota alterations present in BP may play a role in the disease, and certain key microbes and functions may contribute to the link between gut dysbiosis and BP disease activity. Further studies to investigate the underlying mechanisms of the gut-skin interaction are thus clearly warranted, which could aid in the development of potential therapeutic interventions.

## Introduction

Bullous pemphigoid (BP) is the most common autoimmune blistering disorder in Europe, with an incidence of 0.0419 per 1000 person-years, which is increasing by time, and typically develops in individuals over the age of 70 years (1–5). It occurs when autoantibodies target the hemidesmosomal proteins BP180 (type XVII collagen) and/or BP230 in the epidermal basement zone (1, 6, 7). Moreover, BP is characterized by subepidermal blistering and is associated with a significantly decreased quality of life, numerous comorbidities, and a significantly increased mortality risk (6). Thus, the need to further understand the pathogenesis and factors associated with the initiation and progression of BP is instrumental to improve the lives of patients suffering from this disease.

Recently, the host microbiota is considered as a key factor affecting host skin health (8–10). The skin microbiome plays a curial role in the maintaining the skin homeostasis, defending the invasion of pathogens and modulating the immune system (11). There is evidence that microbiota composition of lesional skin, in diseases such as atopic dermatitis and psoriatic disease, show distinct differences compared to healthy skin (8, 12–16). Moreover, these alterations are considered to be involved in the development of several inflammatory dermatoses, which is illustrated e.g. by the predominance of *Staphylococcus aureus* in patients with atopic dermatitis before an outbreak (11). Similarly, studies have indicated a crucial role of skin microbiota in the pathogenesis of BP (17, 18). Of those, one large-scale study involving 228 BP patients thoroughly investigated the skin microbial indicators of BP, in which the abundance of *S. aureus*, an inflammation-promoting species, was reported to be increased in BP patients, suggesting a pathogenic role (17).

Apart from the skin microbiota, the role of gut microbiome in host skin health and disease is also gaining interest. Recent studies revealed intriguing links between gut microbiota and skin diseases (9, 19–22). Firstly, some dermatoses pose as comorbidities of gastrointestinal disorders. For example, 7–11% of patients with inflammatory bowel disease (IBD) also suffer from psoriasis (9). Moreover, multiple inflammatory skin disorders including psoriasis (19, 20, 23–25) and atopic dermatitis (26, 27), are accompanied by gut dysbiosis with altered diversity and composition of the gut microbiota. Decreased levels of *Faecalibacterium prausnitzii* and *Akkermansia muciniphila* in the gut were observed in patients with psoriasis (23, 24, 28), while *Escherichia coli* was reported to be increased in patients with both psoriasis (24) and atopic dermatitis (27). Similarly, a recent study thoroughly characterized the alterations in the gut microbiota in pemphigus, another autoimmune bullous disease, whereby potentially pathogenic bacteria such as *E. coli* and *Bacteroides fragilis* were enriched in patients, while other anti-inflammatory and butyric acid-producing bacteria were significantly reduced (29). On the other hand, gut dysbiosis could increase host vulnerability and trigger an immunological response, resulting in skin imbalances (21, 30). This intricate, bidirectional link known as the “gut-skin axis”, is also thought to involve microbial metabolites such as short chain fatty acids (SCFAs) and gamma-aminobutyric acid (GABA), with SCFAs enhancing epithelial barrier function and GABA inhibiting itch-signaling (30).

The gut microbiota in patients with BP, however, remains largely unexplored, with only a single study to date, with a small sample size and no functional shotgun sequencing data (31). To further investigate the composition and function of the gut microbiota in BP patients, and explore possible interaction between skin conditions and gut microbiota, we thus here conducted a larger study with 16S rRNA gene sequencing and functional shotgun metagenomic sequencing data of 132 individuals. By comparing 66 BP patients and their age-, sex-, and study center-matched controls, we reveal a decreased community richness, altered beta diversity, as well as microbial and functional indicators for disease in BP patients, including *F. prausnitzii*, *Flavonifractor plautii*, and GABA metabolism-related pathways. We furthermore sub-grouped the patients and their corresponding controls according to their disease status (firstly diagnosed and relapsed BP), which reveals similar trends in both clinical conditions.

## Materials and methods

### Ethics statement

All procedures involving human subjects were conducted in accordance with the standards of the ethical policies and procedures approved by the ethics committee of the University of Lübeck (Approval no. 15–051, 18–046), and the respective committees of the study centers, adhering to the Declaration of Helsinki. All participants provided written, informed consent.

### Participant recruitment

A total of 66 pairs of patients with BP and age-, sex-, and study center-matched controls (CL) with non-inflammatory skin diseases such as basal cell carcinoma or squamous cell carcinoma were included in this study. Participant pairs were recruited from seven study centers across Europe (Germany: Dresden (n=14), Freiburg (n=12), Kiel (n=3), Lübeck (n=7) and Würzburg (n=22); Finland: Oulu (n=3); Bulgaria: Sofia (n=5)) from June 2015 to July 2020. All participants were of European descent. Inclusion criteria of BP patients required a diagnosis according to national and international guidelines as described before (17), and no topical antiseptics usage within the prior two weeks and no antibiotic therapy four weeks prior to sampling. Patients can be further grouped into two sub-classifications according to their disease status, firstly diagnosed (BPF) and relapsed ones (BPR). Exclusion criteria for controls included the presence of inflammatory/infectious dermatoses and systemic antibiotics usage within the last four weeks as well. Metadata of BP patients and their controls are provided in **Supplementary Table S1** and summarized demographics are provided in **Supplementary Table S2**.

### Sample collection

Patient and control fecal samples were collected at each study center and immediately frozen at -80°C until further use. Specifically, fecal sampels were transferred with sterile CE-certified plastic spoons into sterile plastic containers (Sarstedt, Numbrecht, Germany) from stool not touching the collection container. DNA extraction from stool samples was performed with QIAamp PowerFecal DNA Kit (Qiagen) following the manufacturer’s instructions. Briefly, a pea-size aliquot of the sample was resuspended in 750 μl of PowerBead Solution and moved to a sterile bead tube with a wide bore tip. Final elution volume was 100 μl and DNA was stored at -20°C until further use. DNA was diluted 1:10 before library preparation.

### 16S rRNA and data processing

The V1-V2 region of the 16S rRNA gene was amplified using 27F and 338R primers using a dual barcoding approach as described in our previous analysis of the skin microbiota in BP (17). The resulting library was sequenced on an Illumina Miseq sequencer (250PE). During the demultiplexing process, only barcodes with no mismatches were allowed (Casava, Illumina). Raw 16S rRNA gene sequencing data were processed using QIIME2 (v2022.8) (32). Specifically, sequences were firstly denoised by the embedded DADA2 (33) for trimming the first 7 or 9 bases from 5’ end of forward or reverse read sequences, truncating the 230 or 200 bases from 3’ end of forward or reverse sequences, and reads were also truncated at the first instance of a quality score smaller than or equal to 3. An abundance table of 16S rRNA amplicon sequence variants (ASVs) was generated, and the taxonomic annotation of ASVs was obtained using the SILVA 138 database (34). To normalize sequencing depth, the samples were randomly sub-sampled to 11,000 sequences. Subsequently, a total of 3,716 ASVs were identified and included in downstream analyses.

### Metagenomic shotgun sequencing and data profiling

Paired-end metagenomic shotgun sequencing was performed on the Illumina NovaSeq platform. Raw metagenomic sequencing reads were subjected to quality control by KneadData v0.12.0 (https://github.com/biobakery/kneaddata), with usage of trimmomatic 0.39 (35) to filter and remove reads with low-quality and reads aligned to the host genome. The clean reads were then aligned to the database PlusPFP (v.20220908, https://benlangmead.github.io/aws-indexes/k2) using Kraken v2 2.1.2 (36, 37), and the taxonomic information at the species level was obtained with Bracken v2.8 (38). The eukaryotic reads (taxid 2759 in NCBI) in clean data were removed using report files produced by Kraken, and the functional profiling was conducted using the Uniref90 protein database in HUMAnN v3.6 (39) with concatenated paired fastq files. This yielded 2,938 species and 417 pathways that were used for downstream analyses.

### Microbial diversity

Analyses of diversity indices were conducted in the R package vegan (R version: 4.2.2; vegan version: v2.6-4). Alpha diversity was assessed by the Chao1 index and Shannon indices, and differences between groups were determined by a non-parametric Wilcoxon test. Statistical analyses of beta diversity based on Bray-Curtis dissimilarity were performed using a non-parametric multivariate analysis (PERMANOVA) (40), which accounts for the effects of confounding factors, including study centers, sex, and age, on microbiome composition. The test was conducted by using the “adonis” function, and the empirical p value was obtained by running 9,999 permutations. When appropriate, statistical p values were adjusted by the Benjamini-Hochberg procedure. For species detected by Kraken and Bracken in the shotgun data, samples were randomly sub-sampled to 1,015,546 reads for diversity analysis.

### Disease-associated gut microbial feature identification

To identify candidate gut microbial features associated with BP, we first performed quality control filtering for taxonomic and functional features. Specifically, an ASV, a species or a pathway needed to have a minimum prevalence of 25%, and minimum relative abundance of 0.01% in at least 10% samples. Gut microbial features associated with BP disease were then determined using linear discriminant analysis effect size (LEfSe) analysis (41), with an absolute value of LDA score no less than 2, and a p value <0.05. The Multivariate analysis by linear models (MaAsLin2) (42, 43) was then applied to evaluate associations between BP disease status and microbial features, correcting for the effect of study center, sex and age, using the Maaslin2 package (v1.12.0) in R. The arcsine square root transformation was performed on relative abundances, and the centred log ratio normalization method in the “Maaslin2” function was performed. Following a similar approach to Kong et al. (43), we considered the intersection of results of LEfSe and MaAsLin2 analyses (abs (LDA) >2, and p value <0.05 in LEfSe analysis; and p value < 0.05 in MaAsLin2 analysis) as criteria for identifying differential microbial features in BP patients.

The Bullous Pemphigoid Disease Area Index (BPDAI) is a validated scoring system commonly used to evaluate the severity of BP disease (17). It takes into account disease activity assessments on both the skin and mucosal surfaces. To evaluate potential correlations between microbial features and BP disease severity, we used the BPDAI scores that were available for a subset of BP patients (44 out of 66). Spearman correlations, corrected for study center, were computed using the “cor.test” function in R. To avoid the influence of zero-inflation, only species or pathways with a prevalence of at least 50% were selected to be included in the correlation analysis. When appropriate, statistical p values were adjusted by the Benjamini-Hochberg procedure.

## Results

**Figure 1:**
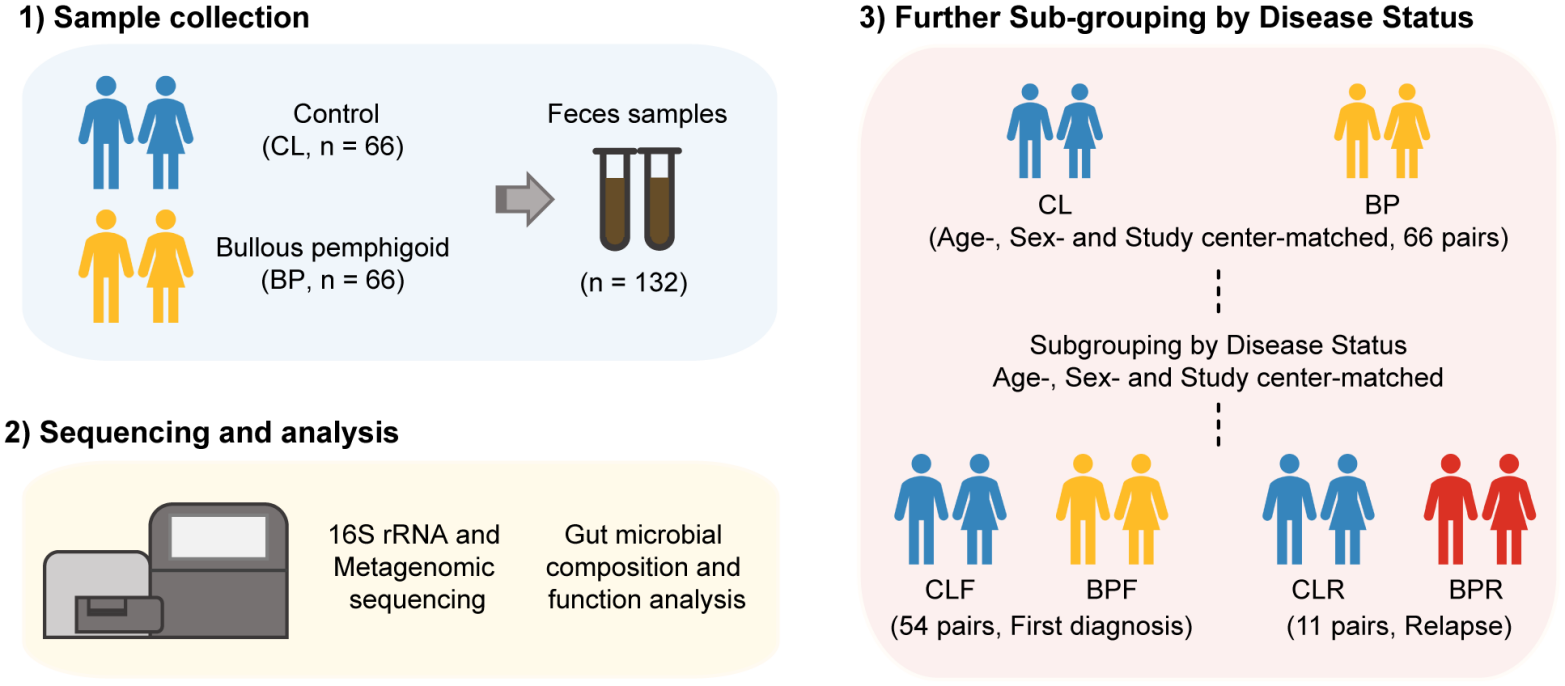
Workflow of investigation on gut microbiome in Bullous pemphigoid (BP) disease. Feces samples were collected from 66 BP patients and their age-, sex- and study center-matched controls (CL). 16S rRNA gene and metagenomic sequencing was then performed to get the profile of gut microbiome of BP patients. The participants were next sub-grouped by disease status of BP patients, that is, firstly diagnosis (CLF and BPF) and relapse (CLR and BPR).

A total of 66 pairs of patients with BP and their matched controls (CL) were recruited from seven study centers located in Germany (5/7; Würzburg, Dresden, Freiburg, Lübeck, and Kiel), Finland (1/7; Oulu), and Bulgaria (1/7; Sofia) between June 2015 and July 2020 (**Tables S1**, **S2**). Of the 66 BP patients, 54 (81.8%) were firstly diagnosed (BPF), 11 (16.7%) had relapsed (BPR), and one (1.5%) was unspecified. The average age of BP patients was 80.26 years (range: 63-98), while that of the 66 controls was 80.64 years (range: 62-100). Of the BP patients and controls, 66.7% were males and 33.3% were females (**Tables S1**, **S2**). Fecal samples were collected from BP patients and their corresponding controls, and bacterial genomic DNA was extracted from the samples. Subsequently, 16S rRNA gene sequencing and shotgun sequencing were conducted (**Figure 1**). To explore the potential impact of disease status on gut microbiota, the BP patients and their corresponding HC were sub-grouped into those who were firstly diagnosed and their matched controls (BPF and CLF) and those who had experienced relapses and their controls (BPR and CLR) (44) (**Figure 1**).

### Decreased alpha diversity and altered overall gut microbial composition in BP patients

**Figure 2:**
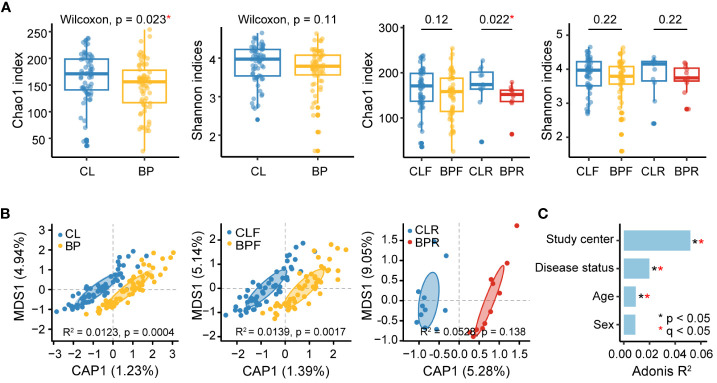
Diversities of gut microbiome of BP patients and their matched controls at the ASV level. **(A)** Alpha diversity of gut microbiome in BP, BPF, BPR and their controls. Abundances of amplicon sequence variants (ASVs) were computed and alpha diversity was assessed by richness (Chao1index) and evenness (Shannon indices). Difference between groups was measured by non-parametric Wilcoxon test. **(B)** Principal coordinates analyses (PCoA) of gut microbiome composition of BP, BPF, BPR groups and corresponding controls (CL, CLF, and CLR) by Bray-Curtis dissimilarity. Statistical significance of beta diversity difference between groups was computed by PERMANOVA in “adonis” function. **(C)** Effect size (Adonis R^2^) of confounding factors and disease status significantly associated with gut microbial variations (Bray-Curtis dissimilarity, PERMANOVA). Statistical p values were adjusted by Benjamini-Hochberg procedure. The “*” means statistical significance based on nominal p value and the “*” in red refer to an adjusted p value (q) smaller than 0.05.

We firstly utilized 16S rRNA gene sequencing data to investigate both alpha and beta diversity in BP patients at the level of amplicon sequence variants (ASVs), which were rarefied to 11,000 reads per sample. Alpha diversity was then assessed with Chao1 index and Shannon indices, which provide measure of richness and evenness of the microbiome, respectively. A significant decrease of the Chao1 index is observed in BP patients, while only a trend towards lower values of the Shannon index is observed (**Figure 2A**). Following the aforementioned sub-grouping, a significant decrease in the Chao1 index is also observed in BPR compared to the CLR group (**Figure 2A**).

Subsequently, beta diversity was analyzed based on Bray-Curtis dissimilarity, and a partial constrained principal coordinate analysis (PCoA) was conducted to examine clustering according to disease status (**Figure 2B**). This reveals a significant difference between BP patients and their controls (adonis R^2 =^ 0.0123, p =0.0004), as well as in a comparison of firstly diagnosed BP patients and their controls (adonis R^2 =^ 0.0139, p = 0.0017). No significant difference is observed between relapsed BP patients and their corresponding controls (adonis R^2 =^ 0.0528, p = 0.138), which may be attributed to sample size limitations. We next evaluated the extent to which confounding factors such as study center, age, and sex contributed to variance in microbial diversity. In line with the previous reports, our analysis indicates that microbial composition is significantly impacted by study center (44), in addition to disease status and age (PERMANOVA q value < 0.05) (**Figure 2C**). A parallel analysis was performed using shotgun data, which, consistent with the findings in 16S rRNA sequencing data, reveals a reduction in the Chao1 index and significant differences in beta-diversity according to disease status (**Figure S1**).

### Disease-associated gut microbes in BP patients

Following the observation of altered patterns of diversity in BP patients, we performed analyses to identify individual taxa contributing to these patterns, both in the 16S rRNA gene and shotgun data. We first conducted linear discriminant analysis effect size (LEfSe) analysis (41) to identify differentially abundant microbial signatures in BP patients, as well as the two BP sub-groups. Multivariate association with linear models (MaAsLin2) (42) was then applied to control for potentially confounding factors, including study center, age and sex. The intersection of significantly differential features identified by LEfSe and MaAsLin2 were deemed as credible BP-associated gut microbial features, following similar previous studies [(43); see Methods]. Compared to the controls, a total of four ASVs are significantly more abundant in BP patients, whereas seven ASVs are significantly reduced (**Figures S2A, B**). Of the increased ASVs, ASV_219, is enriched in relapsed BP patients as well (**Figures S2D, E**), which primarily matches the genus *Flavonifractor* in the SILVA 138 database (34), and *Flavonifractor plautii* when queried in NCBI (E = 2e-154). Conversely, two out of seven ASVs reduced in BP groups belong to *Faecalibacterium*, together with one closely matching *Sutterella* (**Figures S2A, B**). Notably, ASVs matching *Faecalibacterium* are also reduced in both subgroups of BP patients (**Figures S2C-E**). Altogether, alterations in the genera *Flavonifractor* and *Faecalibacterium* appear to be associated with BP disease.

**Figure 3:**
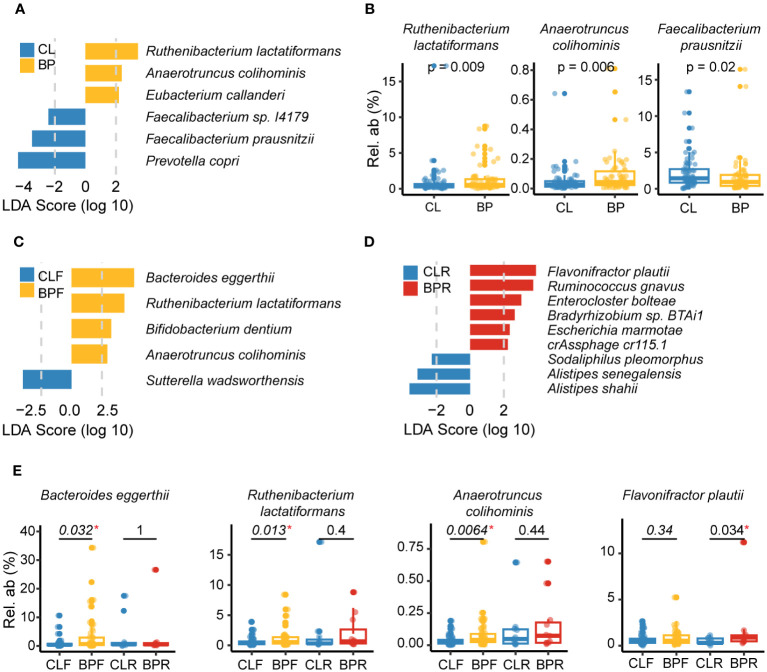
Gut microbes altered in BP disease at the species level. **(A)** Differential species identified by combination of linear discriminant analysis effect size (LEfSe) analysis and Multivariate analysis by linear models (MaAsLin2) in comparison of BP and CL groups based on the metagenomic sequencing. Specifically, the species with an absolute value of LDA score >2 and p <0.05 (calculated by non-parametric Wilcoxon test) in LEfSe analysis was identified as candidates of differential species, and MaAsLin2 was then applied to adjusted effect of study center, age and sex (p <0.05). The blue bars show the bacterial taxa with decreased relative abundance in BP group and the yellow ones refer to those species enriched in BP group after adjusting for the study center using MaAsLin2. **(B)** Representative species that were significantly changed in BP group. The P values were computed by non-parametric Wilcoxon test. **(C, D)** Differential species detected by comparing **(C)** BPF and their matched controls (CLF), or **(D)** BPR and their controls (CLR). Bars with different colors represent altered species in different groups. **(E)** Representative species that were significantly altered in firstly diagnosed or relapsed BP patients (BPF or BPR). The P values were computed by non-parametric Wilcoxon test. “*” means statistically significant in non-parametric Wilcoxon test.

Next, we performed the same differential analyses using shotgun data at the species level. This reveals three species to be enriched in BP patients: *Ruthenibacterium lactatiformans*, *Anaerotruncus colihominis*, and *Eubacterium callanderi*. In contrast, *Prevotella copri*, *Faecalibacterium prausnitzii* and *Faecalibacterium* sp. *I417* are decreased in BP patients (**Figures 3A, B**). Of the three increased in BP, *R. lactatiformans* and *A. colihominis* are more abundant in BP patients who were newly diagnosed (BPF), along with *Bacteroides eggerthii* and *Bifidobacterium dentium* (**Figures 3C, E**). Conversely, *Sutterella wadsworthensis* was found to be decreased in the BPF group. The differential analysis between individuals with relapsed BP disease (BPR) and their controls (CLR) identifies a significant increase in six bacterial species, including *F. plautii*, along with a reduction in the abundance of three other species, such as *Alistipes shahii* (**Figure 3D**). Overall, the observed changes in gut microbial species, including *F. prausnitzii*, *F. plautii*, *R. lactatiformans*, and *A. colihominis*, are likely associated with BP. Notably, *F. plautii* appears to have a more prominent association with BP disease relapse, which is consistent with the findings from the 16S rRNA gene data.

### Functional alterations in the gut microbiome of BP patients

**Figure 4:**
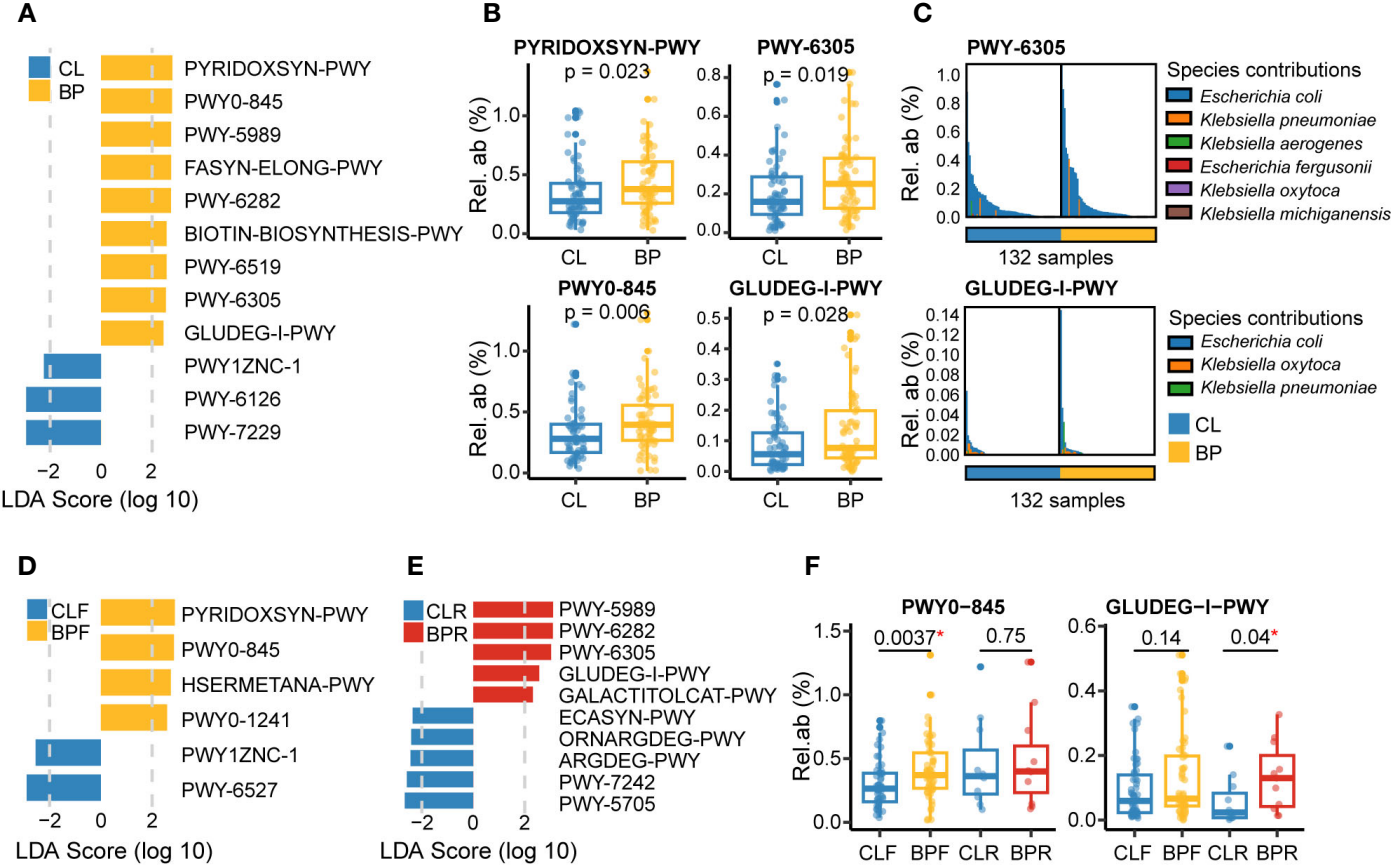
Gut microbial functions altered in BP disease based on the metagenomic sequencing. **(A)** Differential metagenomic pathways altered in BP patients versus their matched control (CL), which were identified by LEfSe analysis (absolute value of LDA score >2 and p <0.05; p values were calculated by non-parametric Wilcoxon test), with MaAsLin2 correcting effect of study center, age and sex (p <0.05). **(B)** Relative abundance of four representative pathways that were significantly changed in BP group. The P values were computed by non-parametric Wilcoxon test. **(C)** Dominant microbial species contributing to two representative pathways: PWY-6305 and GLUDEG-I-PWY, referring to superpathway of putrescine biosynthesis and GABA shunt, respectively. Bars with different colors means different species. **(D, E)** Differential gut microbial functions detected in the comparisons of **(D)** BPF versus their controls (CLF), or **(E)** BPR versus their controls (CLR). **(F)** Relative abundance of two representative pathways that were significantly altered in firstly diagnosed or relapsed BP patients (BPF or BPR). The PWY0-845 means superpathway of pyridoxal 5’-phosphate biosynthesis and salvage. The P values were computed by non-parametric Wilcoxon test. “*” means statistically significant in non-parametric Wilcoxon test.

To investigate differences in the functional potential in the gut microbiome of BP patients, we used the HUMAnN3 workflow [(39); see Methods], followed by the same differential analyses that were applied to the taxonomic data described above. A total of twelve gut microbial pathways are significantly altered in relation to BP. Among the nine pathways more represented in BP groups, two are related to pyridoxal 5’-phosphate biosynthesis [PYRIDOXSYN-PWY (pyridoxal 5’-phosphate biosynthesis I), PWY0-845 (superpathway of pyridoxal 5’-phosphate biosynthesis and salvage)]; three involve fatty acid biosynthesis, [PWY-5989 (stearate biosynthesis II (bacteria and plants)), FASYN-ELONG-PWY (fatty acid elongation – saturated), and PWY-6282 (palmitoleate biosynthesis I (from (5Z)-dodec-5-enoate))]; two are related to biotin biosynthesis [BIOTIN-BIOSYNTHESIS-PWY (biotin biosynthesis I) and PWY-6519 (8-amino-7-oxononanoate biosynthesis I), with amino-7-oxononanoate serving as a biotin precursor (https://metacyc.org)]. The remaining two are PWY-6305 (superpathway of putrescine biosynthesis) and GLUDEG-I-PWY (GABA shunt). In contrast, two pathways in relation to adenosine nucleotides biosynthesis [(PWY-6126 and PWY-7229, superpathway of adenosine nucleotides *de novo* biosynthesis II and I), and PWY1ZNC-1 (assimilatory sulfate reduction IV)] are reduced in BP patients (**Figures 4A, B**).

Notably, the pyridoxal 5’-phosphate serves as a critical cofactor for converting glutamate to GABA (45), which can be involved in the GABA shunt. GABA shunt is a pathway involving the conversion of L-glutamate into GABA, and next converting GABA into succinate with the presence of α-Ketoglutaric acid, which is then coupled with the tricarboxylic acid cycle (46). Additionally, putrescine, whose production was elevated in BP patients, was reported to be a disrupter of intestinal barrier function, with its oral administration increasing gut permeability in mice (47), and furthermore to have immunoregulatory potential such as increasing anti-inflammatory macrophages in the colon and positively correlating with the proinflammatory chemokine CXCL8 in psoriasis patients (48, 49). Moreover, putrescine metabolism also can be linked with GABA shunt, as it is a potential source of GABA to the brain, pancreas, and bacteria including *E.coli* (50–52). We therefore evaluated gut microbial candidate species contributing to PWY-6305 and GLUDEG-I-PWY in the shotgun metagenomic data using the HUMAnN3 workflow. Interestingly, this analysis identifies *E.coli* as the predominant species associated with these two pathways (**Figure 4C**).

Subsequent comparison between BPF and CLF groups revealed six altered pathways in the newly diagnosed BP patients, including three differential pathways identified in the BP group, namely PYRIDOXSYN-PWY, PWY0-845 and PWY1ZNC-1 (**Figure 4D**). Additionally, the other two pathways which were more represented in the BPF group are HSERMETANA-PWY (L-methionine biosynthesis III) and PWY0-1241 (ADP-L-glycero-&beta;-D-manno-heptose biosynthesis). Conversely, the PWY-6527 (stachyose degradation) is one of two pathways reduced in BPF group. We next compared the abundance of pathways between BPR and CLR groups, and a total of ten differential pathways were identified. Notably, we observed that four out of five pathways enriched in the BP group are also significantly enriched in the BPR group (**Figures 4E, F**). Another enriched pathway is GALACTITOLCAT-PWY (galactitol degradation). Five pathways that were decreased in the BPR group are PWY-5705 (allantoin degradation to glyoxylate III), PWY-7242 (D-fructuronate degradation), ARGDEG-PWY (superpathway of L-arginine, putrescine, and 4-aminobutanoate degradation), ORNARGDEG-PWY (superpathway of L-arginine and L-ornithine degradation), and ECASYN-PWY (enterobacterial common antigen biosynthesis). Among them, ARGDEG-PWY is a pathway involved in GABA (4-aminobutanoate) degradation, and is hence also linked to the GABA shunt.

### BP disease severity-associated gut microbial species and functions

The severity of BP disease is commonly assessed using the BPDAI score, a validated scoring system that incorporates disease activity assessments on both the skin and mucosal surfaces (17). To evaluate the potential relationship between gut microbial features and BP severity, we tested for correlations with BPDAI scores, which were available for a subset of BP patients (44 out of 66). The analysis was limited to species or pathways with a prevalence of 50% or greater. After calculating Spearman’s correlation coefficients and correcting for the effect of study center, we identify 30 species and 49 pathways with nominally significant association to the BPDAI score (p < 0.05) (**Figure S3**). After correction for multiple testing, no association is significant (q value > 0.05), although we considered associations with q values < 0.25 as “possible” candidates. Among these, *E. coli* abundance displays the highest positive correlation with BP disease severity (Spearman’s rho = 0.48, p = 0.001, q = 0.2). In terms of pathways, three out of 49 were negatively correlated with BPDAI scores, namely 1CMET2-PWY (folate transformations III (*E. coli*); Spearman’s rho = -0.39, p = 0.0082, q = 0.15), DTDPRHAMSYN-PWY (dTDP-&beta; -L-rhamnose biosynthesis; Spearman’s rho = -0.39, p = 0.0089, q = 0.16), and PWY-6609 (adenine and adenosine salvage III; Spearman’s rho = -0.35, p = 0.02, q = 0.24). Conversely, PWY-5723 displays the highest positive correlation with BPDAI scores (Rubisco shunt; Spearman’s rho = -0.49, p = 0.00075, q = 0.14), together with the two differential pathways in relation to GABA metabolism, namely the GABA shunt (GLUDEG-I-PWY, Spearman’s rho = -0.36, p = 0.017, q = 0.22), and PWY-6305 (Spearman’s rho = -0.31, p = 0.038, q = 0.28, nominally associated). Overall, the positive correlations between GLUDEG-I-PWY, PWY-6305 and *E. coli* (the aforementioned dominant species involved in the GLUDEG-I-PWY and PWY-6305 pathways) with BP disease severity again suggest a potential link between the GABA shunt and BP.

## Discussion

Our study provides a first thorough analysis of the gut microbiome in BP, at both the taxonomic and functional metagenomic level. We find significant alterations of diversity patterns and promising candidate pathways such as the GABA shunt. These findings are in agreement with previous studies reporting altered microbial diversity in various inflammatory and autoimmune diseases, and in particular represents an additional example of a skin disease associated with an altered gut microbiome (53–58).

Among the significant microbial features identified by 16S rRNA gene analysis, increases in abundance of the genus *Flavonifractor* and a decrease of *Faecalibacterium* are of particular interest. *F. prausnitzii* is a well-known microbiomarker of inflammatory diseases, including Crohn’s disease (59, 60), irritable bowel syndrome (61), multiple sclerosis (44), rheumatic diseases (62), and even other inflammatory skin diseases, such as atopic dermatitis, psoriasis, and another autoimmune bullous disease, pemphigus (19, 21, 29). *F. prausnitzii* possesses anti-inflammatory properties, which could contribute to its potential protective effects in inflammatory diseases (63). A reduction of *F. prausnitzii* was also observed in the shotgun data, which adds further reliability to this result. Similarly, the shotgun data also confirms an increase of *F. plautii* in relapsed BP patients, and *F. plautii* was reported to be enriched in early-onset colorectal cancer (43, 64). It is thus intriguing that the increase of *Flavonifractor* and positive correlation between *Flavonifractor* and circulating inflammatory markers (C5a, IL-6, IL-8, IL-7, IL-1β and IL-21) were reported in pemphigus vulgaris, a subgroup of pemphigus (65). *Flavonifractor* was shown to have the ability to cleave quercetin, which has anti-oxidant and anti-inflammatory properties (66). It is thus possible that increased *Flavonifractor* could contribute to oxidative stress and inflammation in the host. Moreover, investigations on a potential immunoregulatory role of *F. plautii* based on mouse models revealed it to inhibit the Th2 immune response, TNF-α expression and interleukin (IL)-17 signaling, thus, potentially alleviating inflammatory responses in allergic diseases, adipose tissue in obesity, and gut inflammation, respectively (67–69). Given these diverse observations, *F. plautii* may contribute to the dysregulation of the immune system in BP leading to the onset or progression of disease. Further research is needed to fully understand the role of *F. plautii* in BP disease. Two other notable species of interest include *R. lactatiformans* and *A. colihominis*, which are increased in the BP and BPF groups; interestingly, both were reported to be associated with multiple sclerosis (44, 70).

Importantly, our inclusion of shotgun metagenomic sequence data provides critical insight into candidate functional pathways associated with BP. In particular, we identified the GABA shunt and related pathways, two involving the biosynthesis of a key cofactor (pyridoxal 5’-phosphate) in GABA conversion into succinate, and one involving biosynthesis of precursor (putrescine) of GABA, which points towards an underlying importance of the gut microbial GABA shunt in BP pathophysiology. Of note, the key metabolite in this bioprocess, GABA, known as an inhibitory neurotransmitter, has various biological activities, such as antioxidant and anti-inflammation (71), and plays multiple roles in maintaining skin health, including inhibition of itching by acting as an inhibitory neurotransmitter (9, 30), attenuating skin lesions by balancing Th1 and Th2 levels and maintaining skin elasticity by increasing the expression of type I collagen (30). Interestingly, GABA metabolism was also reported to be altered in patients with atopic dermatitis, with degradation being significantly positively associated with reduction of disease severity and GABA biosynthesis from putrescine being positively associated with disease severity (72), which suggests a possible positive association between microbial-derived GABA and atopic dermatitis. Further, GABA or GABA receptor agonists were reported to alleviate induced atopic dermatitis or acute skin inflammation in mouse models (73, 74). In the current study, the GABA shunt was observed enriched in the BP patients, with the increased biosynthesis of a key cofactor in GABA shunt, and increased biosynthesis of a precursor of GABA. However, there is evidence that components and activity of GABA shunt can vary a lot even within the same genus (75), which suggests that more refined profiles on GABA metabolism in the gut microbiome need to be carried out to clearly investigate the changes in GABA, GABA metabolism and signaling in BP.

In addition to the GABA shunt and related pathways, other notable pathways, such as PWY-6282 (palmitoleate biosynthesis I (from (5Z)-dodec-5-enoate)), which is higher in BP, overlaps with a previous report showing its increase in individuals with irritable bowel syndrome (76). In contrast, a study investigating links between the human gut microbiome and inflammatory cytokine production reported anti-inflammatory properties of palmitoleic acid (or palmitoleate) by reducing monocyte-derived cytokines, such as IL-1β, TNFα, and IL-6 (77). Finally, the ADP-L-glycero-β-D-mano-heptose in PWY0-1241 (ADP-L-glycero-&beta;-D-manno-heptose biosynthesis), which is more presented in the newly diagnosed BP patients, can be involved in the involved in the synthesis of bacterial lipopolysaccharides (LPS) (78).

Our study has limitations, including a comparatively small sample size and lack of longitudinal data and metabolome data. Further, our age and sex match controls included individuals with basal cell carcinoma or squamous cell carcinoma, thus, it is possible that differences observed in BP could also reflect elements of this type of cancer in the controls. However, the inclusion of these individuals as controls was motivated by the fact that the basal cell carcinoma and squamous cell carcinoma are mainly epidermal skin cancers, characterized by skin lesions or abnormalities, but only rarely metastasize (79, 80). We also excluded patients with any possible metastases to make sure the cancer was always restricted to the epidermis. Moreover, gut dysbiosis was not reported in patients with these cancers thus far (9), despite an altered gut microbiome being reported in other types of cancer, including melanoma, which in contrast can be characterized by rapid metastasis (81, 82). On the other hand, given that these cancers are so common within the elderly, it can be reasonably argued that they genuinely reflect the gut microbiome of this age group (83, 84).

Despite these limitations, our study contributes to a growing body of evidence of common dysbiotic features in the gut microbiome across inflammatory diseases (i.e. reduced alpha diversity, reduced *F. prausnitzii*, role for GABA-related pathways), including those afflicting the skin, and as such emphasize the importance of the gut-skin axis. Future studies including longitudinal data and experimental preclinical models are thus justified to help establish causality and to test microbiome-based intervention strategies.

## Data availability statement

The original contributions presented in the study are publicly available. This data can be found here: https://ncbi.nlm.nih.gov/ under the BioProject accession number PRJNA954584.

## Ethics statement

The studies involving humans were approved by The ethics committee of the University of Lübeck and respective committees of the study centers, adhering to the Declaration of Helsinki. The studies were conducted in accordance with the local legislation and institutional requirements. The participants provided their written informed consent to participate in this study.

## Author contributions

Conceptualization, CS, ES, SI, and JB. Methodology, XL, NB, AC, NA, JB. Software, XL, AC. Validation, XL, AC, JB. Formal Analysis, XL, AC, NA. Investigation, NA, CC. Resources, NB, SB, KD, SG, RG, MG, CG, AG, CH, MH, FH, BL, LH, SV, CS, ES, SI, JB. Data Curation, XL, NB, AC, NA, CC. Writing – Original Draft Preparation, XL, BH, JB. Writing – Review & Editing, all authors. Visualization, XL, AC, JB. Supervision, JB. Project Administration, NB, ES, SI, JB. Funding Acquisition, CS, ES, SI, JB. Data curation, Writing – original draft, FS. All authors contributed to the article and approved the submitted version.
