# Supplementary material for: The gut microbiome in bullous pemphigoid: implications of the gut-skin axis for disease susceptibility
Source: Front Immunol. 2023 Nov 10;14:1212551. doi: 10.3389/fimmu.2023.1212551 (PMC10668026; doi:10.3389/fimmu.2023.1212551)
Supplement: Supplementary file 1 [file DataSheet_1.docx]

Supplementary Material

The gut microbiome in bullous pemphigoid: implications of the gut-skin axis for disease susceptibility

Xiaolin Liu, Nina van Beek, Aleksa Cepic, Nadia A. Andreani, Cecilia J. Chung, Britt M. Hermes, Kaan Yilmaz, Sandrine Benoit, Kosara Drenovska, Sascha Gerdes, Regine Gläser, Matthias Goebeler, Claudia Günther, Anabelle von George, Christoph M. Hammers, Maike M. Holtsche, Franziska Hübner, Dimitra Kiritsi, Franziska Schauer, Beke Linnenmann, Laura Huilaja, Kaisa Tasanen-Määttä, Snejina Vassileva, Detlef Zillikens, Christian D. Sadik, Enno Schmidt^§,*^, Saleh Ibrahim^§,*^, John F. Baines^§,*^

§ These authors contributed equally to this work

* Correspondence:
John F. Baines
[baines@evolbio.mpg.de](mailto:baines@evolbio.mpg.de)

Saleh Ibrahim

[Saleh.ibrahim@uksh.de](mailto:Saleh.ibrahim@uksh.de)

Enno Schmidt

[Enno.Schmidt@uksh.de](mailto:Enno.Schmidt@uksh.de)

## Supplementary Figures


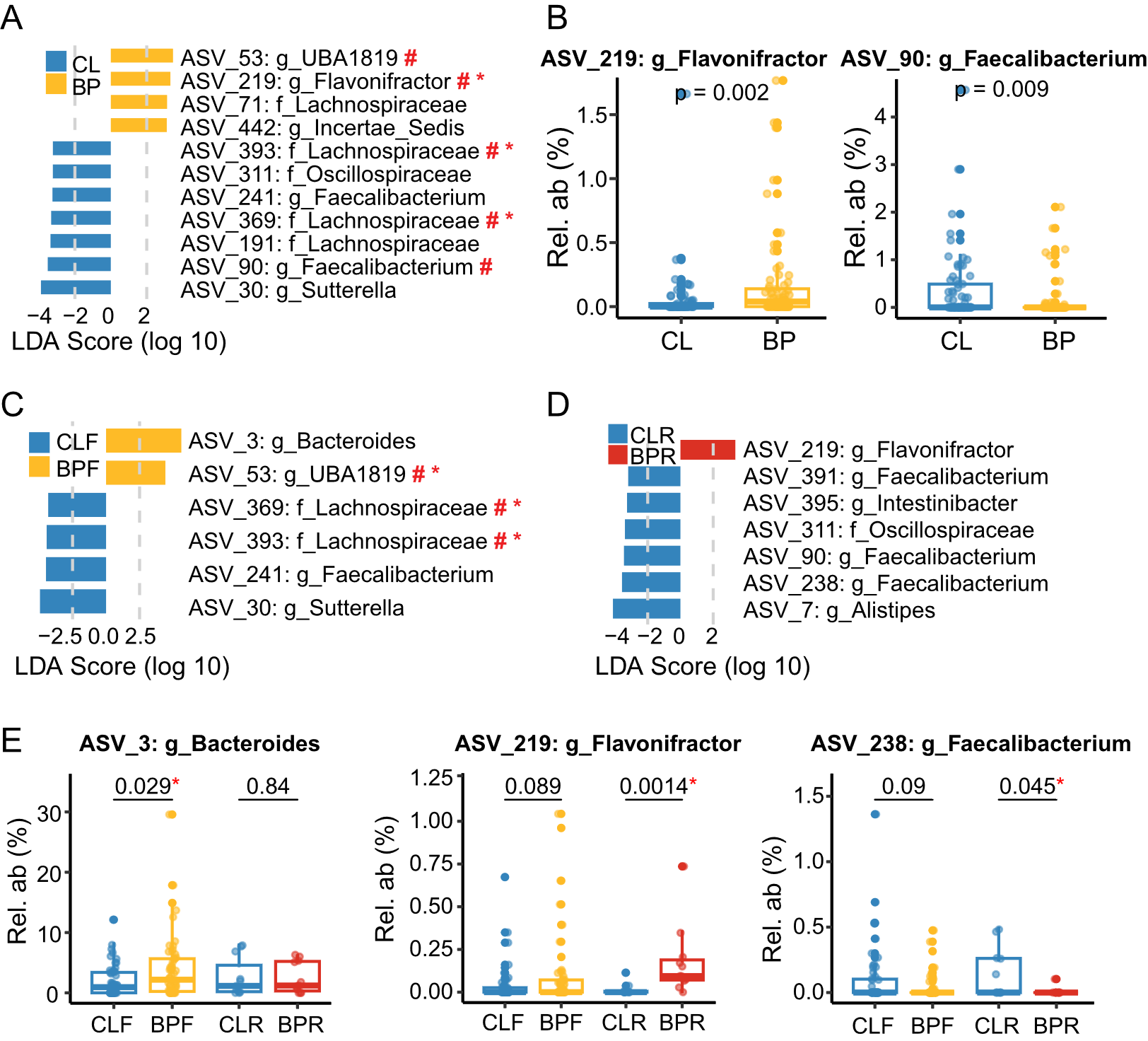


**Supplementary Figure 1.** **Diversities of** **gut microbiome of BP patients and their matched controls at the metagenomic species level.** **(A)** Alpha diversity of gut microbiome in BP, BPF, BPR and their controls (CL). Abundances of species were computed and alpha diversity was assessed by richness (Chao1 index) and evenness (Shannon indices). Difference between groups was measured by non-parametric Wilcoxon test. **(B)** Principal coordinates analyses (PCoA) of gut microbiome composition of BP, BPF, BPR groups and corresponding matched controls (CL, CLF, and CLR) was tested by Bray-Curtis dissimilarity. Statistical significance of beta diversity difference between groups was computed by PERMANOVA in “adonis” function. **(C)** Effect size (adonis R^2^) of confounders and disease status were significantly associated with gut microbial variations (Bray-Curtis dissimilarity, PERMANOVA). Statistical p values were adjusted by Benjamini-Hochberg procedure. The “*” indicates statistical significance based on nominal p value and the “*” in red refers to an adjusted p value (q) smaller than 0.05.


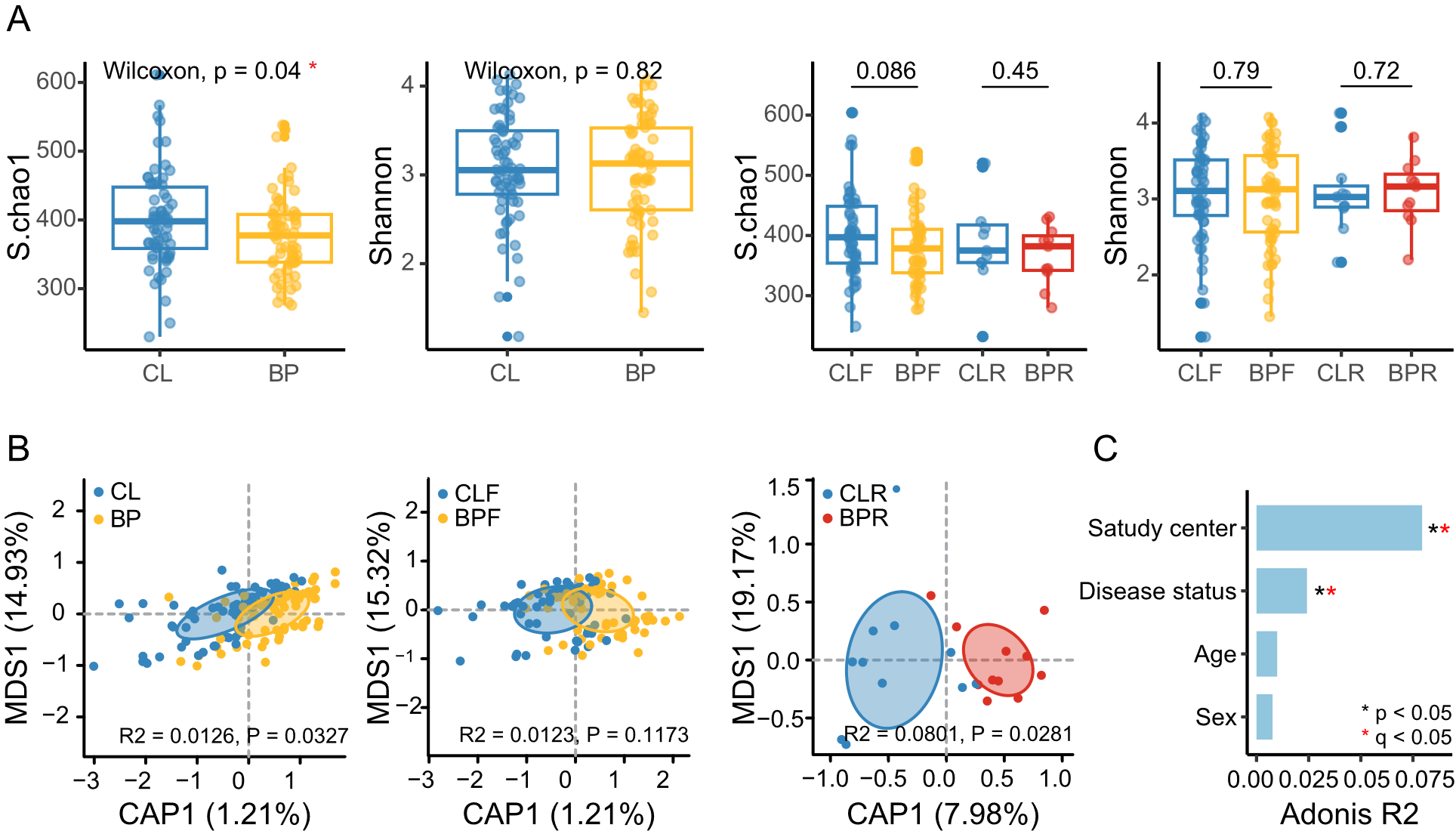


**Supplementary Figure 2. Gut microbes altered in BP disease at the amplicon sequence variant (ASV) level. (A)** Differential ASVs identified by combination of linear discriminant analysis effect size (LEfSe) analysis and Multivariate analysis by linear models (MaAsLin2) in comparison of BP and CL groups based on the 16S rRNA sequencing. Specifically, the ASV with an absolute value of LDA score >2 and p <0.05 (calculated by non-parametric Wilcoxon test) in LEfSe analysis was identified as candidates of differential ASV, and MaAsLin2 was then applied to adjust for effects of study center, age and sex (p <0.05). The blue bars show the ASVs with decreased relative abundance in the BP group and the yellow ones refer to those ASVs enriched in the BP group after adjusting for the study center using MaAsLin2. The ASV marked by “#” has a Benjamini-Hochberg procedure-based adjusted p value smaller than 0.1 in LEfSe analysis. The “*” refer to a Benjamini-Hochberg procedure-based adjusted p value smaller than 0.1 MaAsLin2 workflow. **(B)** Representative ASVs that were significantly changed in the BP group. P values were computed by non-parametric Wilcoxon test. **(C-D)** Differential ASVs detected by comparing **(C)** BPF and their controls (CLF), or **(D)** BPR and their controls (CLR). Bars with different colors represent altered ASVs in different groups. **(E)** Representative ASVs that were significantly altered in firstly diagnosed or relapsed BP patients (BPF or BPR). P values were computed by non-parametric Wilcoxon test.


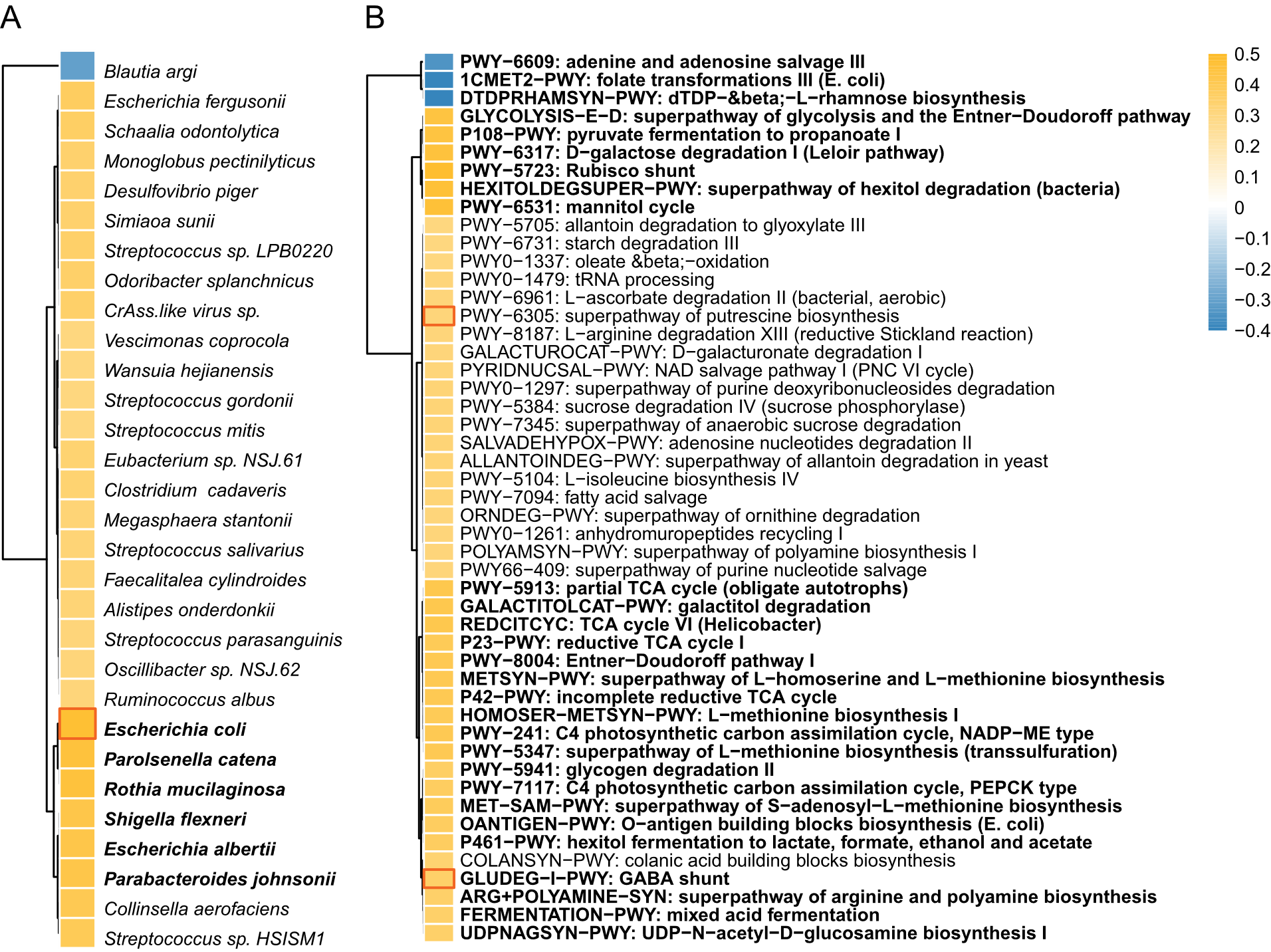


**Supplementary Figure 3. Spearman correlations between Bullous Pemphigoid Disease Area Index (BPDAI) and metagenomic species or pathways. (A)** The Spearman correlations between BPDAI scores and gut species with study center corrected (p.value <0.05) for a subset of BP patients (n = 44). The bold ones are BP disease severity-associated species with adjusted p value (q.value) < 0.25. (B) The Spearman correlations between BPDAI scores and gut microbial pathways with study center corrected (p.value <0.05) for a subset of BP patients (n = 44). The bold ones are BP disease severity-associated pathways with adjusted p value (q.value) < 0.25. The rectangles circled by red lines are species or pathways of particular interest. The species or pathways were filtered by a prevalence of not less than 50% for minimizing the effect of zero-inflation.
